# Energy-Adjusted Dietary Intakes Are Associated with Perceived Barriers to Healthy Eating but Not Food Insecurity or Sports Nutrition Knowledge in a Pilot Study of ROTC Cadets

**DOI:** 10.3390/nu13093053

**Published:** 2021-08-31

**Authors:** Elizabeth Daniels, Jennifer Hanson

**Affiliations:** Department of Food, Nutrition, Dietetics and Health, Kansas State University, Manhattan, KS 66506, USA; eedaniels@ksu.edu

**Keywords:** athletes, dietary intake, eating behaviors, food security, military personnel, nutrition knowledge

## Abstract

Military service is inherently demanding and, due to the nature of these demands, the term “tactical athlete” has been coined to capture the physical requirements of the profession. Reserve Officers’ Training Corps (ROTC) cadets are a unique subset of the military service community, and the complexity of their training and educational pursuits increases their susceptibility to unhealthy eating patterns. The purpose of this pilot study was to explore the relationship between the perceived barriers to healthy eating, food insecurity, sports nutrition knowledge, and dietary patterns among Army ROTC cadets. The usual dietary intake was gathered from (*N* = 37) cadets using the General Nutrition Assessment Food Frequency Questionnaire. The perceived barriers to healthy eating were measured using a set of scales consisting of social barriers (6 items, α = 0.86), access barriers (2 items, α = 0.95), and personal barriers (2 items, α = 0.67), with higher-scale scores indicating greater perceived barriers. Spearman correlation coefficients were used to measure the association between the energy-adjusted dietary intakes and the scores on the barriers scales. Energy-adjusted intakes of calcium (ρ = −0.47, *p* ≤ 0.01), fiber (ρ = −0.35, *p* = 0.03), vitamin A (ρ = −0.46, *p* ≤ 0.01), vitamin C (ρ = −0.43, *p* ≤ 0.01), fruit (ρ = −0.34, *p* = 0.04), and vegetables (ρ = −0.50, *p* ≤ 0.01) were negatively correlated with the perceived personal barrier scores. The energy-adjusted intakes of fiber (ρ = −0.36, *p* = 0.03), vitamin C (ρ = −0.37, *p* = 0.03), and vitamin E (ρ = −0.45, *p* ≤.01) were negatively correlated with perceived social barriers, while energy-adjusted vitamin C intake was negatively correlated with perceived access barriers (ρ = −0.40, *p* = 0.01). Although additional research is needed to better understand the dietary patterns of ROTC cadets, among the participants in this study, greater perceived personal, social, and access barriers were associated with less nutrient-dense eating patterns. Interventions aimed at addressing such barriers may prove beneficial for the improvement of diet quality among ROTC cadets.

## 1. Introduction

The modern soldier is expected to be stronger, faster, and more adaptable than ever before. Tactical training focuses on increasing strength, agility, power, and speed to enhance physical performance [1]. Given the inherently demanding nature of military service, the term “tactical athlete” has been coined to capture the physical requirements of the profession [1]. Although physical performance is a multifaceted outcome, the revisions to *Field Manual (FM) 7–22: Holistic Health and Fitness*, formerly named “Army Physical Readiness Training”, recognize nutrition as one of the five essential pillars of wellbeing that the Department of Defense (DOD) “deems essential to military mission success” [2]. Among military tactical athletes (i.e., Special Forces Assessment and Selection candidates), higher diet quality has been shown to be associated with better physical performance [3]. Additionally, higher diet quality has also been shown to be associated with greater psychological resilience among military recruits [4].

Both the nature of the profession and the requirement to maintain body weight standards makes sound nutrition essential. However, Reserve Officers’ Training Corps (ROTC) cadets are a unique subset of the military service community, and the complexity of their training and educational pursuits increases their susceptibility to unhealthy eating patterns. ROTC cadets are college students who receive branch-specific military training in conjunction with their regular academic studies. Cadets often receive tuition assistance or scholarships in exchange for contracted military service. Military service begins following the completion of their chosen degree, and upon graduation cadets become commissioned officers and leaders in the U.S. Army. ROTC cadets are also a unique subset of the college student population, having to navigate the college environment while also maintaining U.S. Army fitness and body composition standards. Due to multiple obligations, such as regular physical training, academic assignments, the introduction to military procedures, and the expected lifestyle changes that occur in college, cadets are susceptible to poor eating habits, poor body image, and disordered eating [5].

College is often a time of transition, with increased autonomy and responsibility. For many students, this is the first time that they find themselves purchasing and preparing foods independently from a parent or caregiver. This increased freedom and the associated change in lifestyle often lead to poor choices that can increase the risk of nutrition-related chronic health conditions. For example, a recent survey of college students revealed that 65.5% of undergraduate students consumed sugar-sweetened beverages daily while only one-third consumed three or more servings of vegetables per day [6]. In addition to promoting overall physical health, proper nutrition has been found to be positively correlated with academic success in college [7]. As such, nutrition’s role in physical well-being and academic performance makes it important to practice healthy eating habits during college years.

The Dietary Guidelines for Americans, which encourage healthy eating patterns at every stage of life, recommend that individuals meet their food group needs with nutrient-dense foods and beverages while staying within their calorie limits [8]. Unfortunately, the dietary habits of Americans, including those of college students [6], college athletes [9], and service members [10], are less than desirable. Roughly 17% of active-duty military personnel [11] and 16% of undergraduate college students [6] are considered obese. Despite nutritional education initiatives [12] and access to healthcare professionals, active-duty soldiers, regardless of rank, indicate that they feel that their nutrition is “out of their control”, stating that the current environmental and cultural norms of military life discourage healthful choices while encouraging poor ones [13]. Similarly, among college athletes, a lack of time (31.8%), a lack of knowledge (28.6%), and financial restrictions (21.0%) are the most frequently reported obstacles to optimal nutrition [14].

Although research shows that many athletes underestimate their nutrient needs [14] and many also fail to meet the nutrition guidelines for optimal performance [15,16,17], there has been little investigation into the dietary intakes and unique perspectives of ROTC cadets on college campuses. Busy schedules that include work, cadet responsibilities, pressures to maintain good academic standing, and the requirement to meet Army body composition standards make it important to understand the barriers these future Army officers face when making nutritional choices. Therefore, the purpose of this pilot study was to explore the relationship between the potential obstacles to healthy eating and dietary intake during the academic year among Army ROTC cadets. The potential obstacles to healthy eating examined in this study included: perceived barriers to healthy eating, food insecurity, and a lack of sports nutrition knowledge.

## 2. Materials and Methods

### 2.1. Experimental Design and Participants

A cross-sectional design was used to explore the relationship between potential obstacles to healthy eating and dietary patterns among Army ROTC cadets attending a land-grant university in the Midwest region of the U.S. The recruitment of study participants began in February 2021, with researchers visiting ROTC classes. Cadets were given a brief overview of the purpose of the research study and asked for their voluntary participation. Prospective research participants were informed that cadres would not have access to their results or information and that their participation would not interfere with their standing in the program. Incentives to participate were not provided. Of the 87 cadets enrolled in the ROTC program, 37 (42.5%) volunteered to participate. Data collection occurred on two dates in March of 2021, during regularly scheduled physical training time, in a classroom setting. Cadre and cadet command were not involved in the data collection. The cadets were asked to dress in the Army physical training uniform in order to standardize the collection of body weight data. The average time taken to complete the surveys was roughly 60 min. This study protocol was approved by the Kansas State University Institutional Review Board (proposal #10370).

### 2.2. Procedures

A self-administered, paper-based questionnaire was used to obtain demographic characteristics and measure perceived barriers to healthy eating, food insecurity, sports nutrition knowledge, and usual dietary intake. The cadets’ heights and weights were measured and recorded by researchers using the Health-o-Meter portable digital floor scale, model 349KLX (Pelstar, LLC, McCook, IL, USA), and a Seca 217 stadiometer (GmbH & Co. KG, Hamburg, Germany). Body mass index (BMI) was calculated using the recorded heights and weights in the standard mathematical formula: weight (kg)/[height (m^2^)].

### 2.3. Instruments

#### 2.3.1. Perceived Barriers to Healthy Eating

A series of questions regarding barriers to healthy eating were developed from a set of questions used to examine the barriers to weight maintenance [18]. The previously used questions were modified to better capture potential barriers among ROTC cadets. For example, one question from the original study was deleted because it asked about support from children, and two questions were added in order to include the time barriers related to school and ROTC. Response options for all survey questions were: “strongly disagree”, “disagree”, “neither agree or disagree”, “agree”, and strongly agree”. Each option was assigned a value from 1 to 5, with the assigned values increasing as the perceived barriers increased.

#### 2.3.2. Sports Nutrition Knowledge

The Nutrition Knowledge for Young and Adult Athletes (NUKYA) survey was used to measure sports nutrition knowledge. This survey is validated for use in the assessment of team sports athletes and was chosen for its low time burden, updated content, and ease of use. The survey consists of 59 items that assess macronutrients, micronutrients, hydration, and food intake periodicity [19]. The NUKYA was scored by assigning correct answers +1 point, incorrect answers −1 point, and unanswered items 0 points. Higher scores indicated greater knowledge; the maximum number of points possible was 59.

#### 2.3.3. Food Security

The U.S Department of Agriculture (USDA)’s six-item short form module [20] was used to measure food security. The six-item short form module was chosen for its low respondent burden and its high level of reliability in determining food security in populations [21]. Cadets were asked to respond to statements regarding their experiences in the 12 months preceding the study. Survey responses that resulted in a raw score of 0–1 were considered food-secure, and survey responses that resulted in a raw score of 2–6 were categorized as food-insecure.

#### 2.3.4. Dietary Intake

The cadets’ dietary intake over the six months preceding the study was measured using the General Nutrition Assessment Food Frequency Questionnaire (FFQ) developed by the Nutrition Assessment Shared Resource (NASR) of Fred Hutchinson Cancer Research Center, Seattle, Washington. The nutrient calculations were performed using the Nutrient Data System for Research (NDSR) software, developed by the Nutrition Coordinating Center, University of Minnesota, Minneapolis, MN [22]. The FFQ-determined nutrient and food group intakes were adjusted for energy intake and then used as a measure of the cadets’ usual dietary intake during the academic year.

### 2.4. Data Analysis

Data analyses were performed using an SPSS version 27 (IBM, Armonk, NY, USA). Descriptive analyses were performed in order to characterize the sample. The demographic responses were collapsed into meaningful categories (i.e., working vs. not working) to allow comparisons between these categories by body weight category and food security status, using Chi-square tests (Χ^2^). Fisher’s exact test values were examined in instances where the Chi-square expected cell counts were less than five.

For the questions regarding the barriers to healthy eating, principal component analysis and Cronbach’s alpha values were used as an assessment of construct validity and internal consistency, respectively. Shapiro-Wilk tests revealed that many of the diet and perceived barrier values were non-normally distributed. Therefore, nonparametric statistical analyses were used in the analysis of these variables. Spearman rank-order correlation coefficients were used to measure the association between energy-adjusted dietary variables, sports nutrition knowledge scores, and barriers to healthy eating scores. Mann–Whitney U tests were used to compare energy-adjusted dietary variables, sports nutrition knowledge scores, and barriers to healthy eating scores, based on food insecurity status and demographic group category.

## 3. Results

### 3.1. Participants

Thirty-seven ROTC cadets completed the study. The majority were men (70%), and most were White/Caucasian (86.5%). Over two-thirds were enrolled in more than 12 credit hours (72.9%), and a similar proportion were employed, either full-time or part-time (67.6%). The age of the participants ranged from 18 to 33 years, with a mean age of 22.2 years (SD = 3.1). Most participants classified themselves as either a junior (*n* = 13; 32.4%) or a senior (*n* = 15; 40.5%). The mean BMI was 25.7 kg/m^2^ (SD = 2.9), with four (10.8%) cadets having a BMI > 29.9, classifying them as obese. Table 1 contains additional information regarding the participants’ characteristics.

### 3.2. Perceived Barriers to Healthy Eating

#### 3.2.1. Instrument Evaluation

Principal component analysis of the perceived barriers to healthy eating questions resulted in the deletion of two items and the subsequent identification of three multiple dimensions. Following inspection and interpretation of the data, the three dimensions were deemed to be (a) perceived social barriers, (b) perceived access barriers, and (c) perceived personal barriers. Therefore, three summated rating scales were used as operational indicators of the perceived barriers to healthy eating. Cronbach’s alpha values were 0.86, 0.95, and 0.77 for the perceived social barriers scale, the perceived personal barriers scale, and the perceived access barriers scale, respectively.

Median with interquartile range (IQR) scores and response frequencies for the items comprising each scale, as well as for those items not retained, are located in Table 2. The two barrier items with the greatest percentage of agreement responses were, “I do not have enough time to prepare or eat healthy foods because of school”, and, “I do not have time to prepare or eat healthy foods because of my commitment to ROTC”.

#### 3.2.2. Perceived Barriers Scales

The median (IQR) scale scores were 15.0 (1–10) for the perceived social barriers scale, 3.0 (2–4) for the perceived access barriers scale, and 5.0 (3–6) for the personal barriers scale. Table 3 contains the results of the comparisons of perceived barriers scale scores by participant characteristics. The perceived personal barriers scores did not differ significantly based on gender, racial/ethnic category, (i.e., White/Caucasian or all others), employment category, (i.e., working or not), or body weight status (i.e., non-obese or obese). The perceived social barriers scores did not differ significantly based on gender, racial/ethnic category, employment category, or body weight status. Perceived access barriers scores did not differ significantly based on gender, racial/ethnic category, or employment category; however, a trend (*p* = 0.08) was observed with those categorized as obese having higher perceived access barriers scale scores.

### 3.3. Food Insecurity

Twenty-six (70.3%) of the participating cadets were classified as food-secure, ten (27.0%) were classified as food-insecure, and one participant (2.7%) did not complete the food security questions and therefore could not be classified. The food security category did not differ significantly based on gender, *p* = 0.41, Fisher’s exact test; racial/ethnic category, *p* = 1.0, Fisher’s exact test; employment category, *p* = 0.12, Fisher’s exact test; or body weight status *p* = 0.31, Fisher’s exact test.

### 3.4. Sports Nutrition Knowledge

The NUKYA scores ranged from 0 to 45 points. The median (IQR) score was 23 (19.5–26.5) out of 59 possible points (i.e., 38.9% of the maximum possible points). The scores on the nutrition knowledge survey did not differ significantly based on gender (U = 138.5, *p* = 0.88), racial/ethnic category (U = 74.5, *p* = 0.81), employment category (U = 127.0, *p* = 0.47), body weight status (U = 48.0, *p* = 0.41), or food security status (*p* = 0.89).

### 3.5. Dietary Intake

The FFQ-determined nutrient and food group intake values are provided in Table 4. The median (IQR) energy intake was 2174 kcal/day (1664–2786). The median (IQR) energy intake was higher (U = 73, *p* = 0.02) among men at 2414 kcals/day (1951–2901) compared to women at 1767 kcals/day (1512–1938). Energy intake did not differ significantly based on racial/ethnic category (U = 50, *p* = 0.20), employment category (U = 144.0, *p* = 0.86), body weight status (U = 43, *p* = 0.28), or food security status (U = 118, *p* = 0.67).

The correlation coefficients for the dietary intake variables and the perceived barriers, as well as for sports nutrition knowledge, are listed in Table 5. The energy-adjusted intakes for calcium (ρ = −0.47, *p* ≤ 0.01), fiber (ρ = −0.35, *p* = 0.03), vitamin A (ρ = −0.46, *p* ≤ 0.01), vitamin C (ρ = −0.43, *p* ≤ 0.01), fruit (ρ = −0.34, *p* = 0.04), and vegetables (ρ = −0.50, *p* ≤ 0.01) were negatively correlated with the perceived personal barrier scale scores. The energy-adjusted intakes for fiber (ρ = −0.36, *p* = 0.03), vitamin C (ρ = −0.37, *p* = 0.03), and vitamin E (ρ = −0.45, *p* ≤ 0.01) were negatively correlated with the perceived social barriers, while energy-adjusted vitamin C intake was negatively correlated with perceived access barriers (ρ = −0.40, *p* = 0.01). The correlations between the energy-adjusted dietary intakes and the NUKYA scores were non-significant (Table 5).

Table 6 contains the results of the comparisons of the dietary intake variables by participant food security status. The energy-adjusted dietary intakes did not differ significantly based on food security status.

## 4. Discussion

Through this study, an improved understanding of the relationship between potential obstacles to healthy eating and diet quality among Army ROTC cadets has been achieved. In addition, this study provides insight into the healthy eating barriers that cadets face on a regular basis. As revealed in the results, personal, social, and access barriers were associated with less nutrient-dense diets. This finding is consistent with that of a review by Munt et al. [23], in which key barriers to healthy eating among young adults included a lack of motivation, the unhealthy diet of family and friends, the expectation to consume unhealthy foods in some situations, and a lack of time to plan, shop, prepare, and cook healthy foods. Similarly to what was observed among college athletes [14], a lack of time was the most commonly reported barrier in the present study.

These findings suggest that cadets could benefit from information on strategies to overcome these barriers, particularly time barriers. Time pressures may lead to an increased reliance on convenience foods, which are often high in energy, added sugars, saturated fats, and sodium, and low in vitamins and minerals [24,25,26]. The provision of time management strategies and information on foods that can be prepared easily could be of value. Because regular breakfast consumption is linked to higher diet quality among soldiers [27], allowing adequate time for a breakfast meal after early morning physical training and classes could lead to healthier overall eating patterns.

While the barrier item related to the skills needed to “plan, shop for, or cook healthy foods” was not retained following principal component analysis, the barrier conceptualized by this item has been recognized by others [23]. In an earlier study of college students, fruit and vegetable intake was shown to be associated with cooking frequency but not with cooking confidence [28]. Future research is needed to clarify the relationship between these variables among the ROTC cadet population.

Neither food security status nor sports nutrition knowledge were associated with diet quality in the present study. Although Zein et al. [29] reported that food-insecure college students were more likely to experience poor sleep quality, high stress, and disordered eating, the researchers did not find a significant relationship between food insecurity status and nutritional status (i.e., BMI). With the median score equal to 38.9% of total possible points, overall sports nutrition knowledge appears to be low. This finding is similar to what others have observed when measuring sports nutrition knowledge among athletes [14,19].

While the purpose of this study was to look at diet quality, and not specifically BMI, 48% of cadets in this study were found to be overweight or obese based on their BMI. Although BMI is not a direct indicator of health for athletes or military personnel due to the increased lean body mass observed in these populations, research shows that a higher BMI may lead to early dismissal from the ROTC program or service [2,30,31]. Higher BMI and poor nutritional status also contribute to increased healthcare costs from injury and chronic disease, and lost productivity in the military [19,32,33].

The results of this study are timely and pertinent to the military service community, as well as to those who work with this population of tactical athletes. This study captured the nutrient-density of eating patterns during the academic year during a period that spanned from fall through spring. Among military service members, diet quality is associated with physical performance [3,27] and psychosocial health [27]. The strengths of this study include the use of validated surveys to collect information about nutritional knowledge and dietary intake. Average energy intakes of 2409 kcal/day and 1985 kcal/day have been reported for men and women 20 to 29 years old, respectively [34]. Considering these average daily energy values for young adults, the FFQ-determined daily energy intakes in the current study appear to be reasonable. An additional strength is that the anthropometric variable was measured by researchers instead of relying on participant-reported heights and weights. Lastly, the development of the perceived barriers to healthy eating scales provides further information that may be useful in future studies.

A limitation of this study was the relatively small sample size. In addition, while information about the demographic characteristics of Army ROTC cadets does not appear to be available to the public, the proportion of respondents who were female (29.7%) and the proportion who self-identified as White/Caucasian (86.5%) were higher than the proportion of Army officers who are female (17.9%) and the proportion of Army officers who self-identify as White (73.4%) [35]. Lastly, the majority of the cadets in this study were either juniors or seniors (72.9%). Having a larger sample of upperclassmen may have skewed results because this group of students have time both to acclimate themselves to the college routine and to learn to balance commitments such as school, work, and ROTC. Future studies should be conducted among a larger and more representative sample, and should include measures of diet quality in addition to nutrient density.

## 5. Conclusions

Cadets in the ROTC are faced with multiple transitional periods that may contribute to making poor dietary choices. In this particular study, it was determined that obstacles to healthy eating exist and that obligations to friends, family, school, work, and the ROTC may all contribute to poor nutritional outcomes and an increased risk of injury, fatigue, and chronic health conditions. For future military leaders, the early formation of healthy habits is imperative for career progression, the prevention of injury, the deterrence of early separation from service, and an overall reduction in healthcare costs. Cadets in the ROTC could benefit from instruction and guidance on how to overcome these barriers. The inclusion of evidence-based information, support, and local resources through individual programs within the curriculum may help to support a cadet’s ability to make healthful food choices. Opportunities exist for additional research into the barriers cadets face to living a healthy lifestyle, as well as into the development of tools to reduce these barriers. Research suggests that there is a need for longitudinal studies examining cadets across multiple branches and program locations, in order to better serve cadets from their entry into ROTC through the first years of their professional military service.

## Figures and Tables

**Table 1:** Participant characteristics (*N* = 37).

Characteristic	Category	*n* (%)
Gender	Male	26 (70.3)
	Female	11 (29.7)
Race/Ethnicity	Asian/Pacific Islander	1 (2.7)
	Black/African American	2 (5.4)
	Hispanic/Latino	1 (2.7)
	White/Caucasian	32 (86.5)
	Multi/Biracial	1 (2.7)
Age	≤23 years	27 (83.8)
	≥24 years	10 (73.0)
BMI	≤24.9	18 (48.6)
	25.0–29.9	15 (40.5)
	≥29.9	4 (10.8)
Student Classification	Freshman	3 (8.1)
	Sophomore	6 (18.9)
	Junior	13 (32.4)
	Senior	15 (40.5)
Credits Enrolled	>12	27 (73)
	8–12	9 (24.3)
	<8	1 (2.7)
Work Status	Full time > 35 h/week	7 (18.9)
	Part time < 35 h/week	18 (48.6)
	Not working	12 (32.4)

**Table 2:** Perceived barriers responses.

Barriers to Healthy Eating	Median (IQR) *	Strongly Disagree (%)	Disagree (%)	Neither Agree/Disagree (%)	Agree (%)	Strongly Agree (%)
Social barriers (α = 0.86)						
I do not have family support to eat a healthy diet	2.0 (1.5–3.0)	16 (43.3)	9 (24.3)	7 (18.9)	4 (10.8)	1 (2.7)
I do not have the support from friends to eat a healthy diet	2.0 (1.0–2.5)	15 (40.5)	13 (35.1)	5 (13.5)	4 (10.8)	-
I do not have time to prepare or eat healthy foods because of my job	2.0 (1.5–3.0)	10 (27.0)	9 (24.3)	10 (27.0)	5 (13.5)	3 (8.1)
I do not have time to prepare or eat healthy foods because of family commitments	2.0 (1.0–2.0)	14 (37.8)	18 (48.6)	3 (8.1)	2 (5.4)	-
I do not have enough time to prepare or eat healthy foods because of school	3.0 (2.0–4.0)	6 (16.2)	9 (24.3)	6 (16.2)	12 (32.4)	4 (10.8)
I do not have time to prepare or eat healthy foods because of my commitment to ROTC **	3.0 (2.0–4.0)	8 (21.6)	9 (24.3)	6 (16.2)	13 (35.1)	1 (2.7)
Access barriers (α = 0.95)						
I do not have access to healthy foods	2.0 (1.0–2.0)	18 (48.6)	13 (35.1)	3 (8.1)	3 (8.1)	-
I am unable to buy healthy foods	1.0 (1.0–2.0)	19 (51.4)	14 (37.8)	2 (5.4)	2 (5.4)	-
Personal barriers (α = 0.77)						
I do not have enough information about a healthy diet	2.0 (1.5–3.0)	9 (25.3)	18 (48.6)	2 (5.4)	6 (16.2)	2 (5.4)
I do not have the motivation to eat healthy	2.0 (2.5–3.0)	5 (13.5)	16 (43.2)	8 (21.6)	7 (18.9)	1 (2.7)
Not retained						
I do not enjoy eating healthy foods	2.0 (1.0–3.0)	12 (32.4)	19 (51.4)	2 (5.4)	3 (8.1)	1 (2.7)
I do not have the skill to plan, shop for, or cook healthy foods	2.0 (1.0–3.0)	13 (35.1)	13 (35.1)	3 (8.1)	4 (10.8)	4 (10.8)

* Interquartile range, ** Reserve Officers’ Training Corps.

**Table 3:** Comparisons of perceived barriers scale scores by participant characteristics.

Characteristics		*n*	Social Barriers		Access Barriers		Personal Barriers	
			U	*p* *	U	*p* *	U	*p* *
Gender	Male	26	134.0	0.78	125.5	0.57	105.5	0.22
	Female	11						
Race/ethnicity	White/Caucasian	32	69.0	0.65	72.5	0.75	56.0	0.31
	All others	5						
Employment	Working	25	150.0	1.0	135.0	0.64	135.5	0.64
	Not Working	12						
Body weight status	Non-Obese	33	62.0	0.87	30.0	0.08	45.0	0.33
	Obese	4						

* Based on the Mann–Whitney U test using exact significance values.

**Table 4:** Food Frequency Questionnaire-determined daily nutrient and food group intakes (*N* = 37).

Nutrient/Food Group	Mean	Median (IQR)
Energy (kcal)	2393	2174 (1664–2786)
Added sugar (g)	59.4	45.5 (29.7–70.5)
Calcium (mg)	1063.6	1019.6 (700.7–1251.0)
Fiber (g)	20.3	17.4 (14.1–24.1)
Folate (mcg)	495.7	442.7 (323.7–613.3)
Iron (mg)	15.6	14.6 (10.0–18.6)
Saturated Fat (g)	33.1	18.8 (20.3–39.6)
Sodium (mg)	4205.4	3887.6 (2984.2–4673.3)
Vitamin A (mcg)	996.2	693.8 (573.1–1020.2)
Vitamin B12 (mcg)	7.0	6.0 (3.8–8.1)
Vitamin C (mg)	109.2	86.7 (55.1–147.4)
Vitamin E (IU)	20.3	16.4 (12.8–24.8)
Whole Grains (oz eq)	1.9	1.4 (1.0–2.7)
Fruit	1.0	0.9 (0.5–1.1)
Vegetable	1.8	1.2 (0.9–2.80)

**Table 5:** Correlations between dietary intakes and perceived barriers, as well as sports nutrition knowledge *(N* = 37).

Energy-Adjusted Dietary Intake	Personal Barriers	Social Barriers	Access Barriers	Sports Nutrition Knowledge
Added Sugar	0.06	0.08	0.07	−0.13
Calcium	−0.47 **	−0.27	−0.07	0.22
Fiber	−0.35 *	−0.36 *	−0.32	0.06
Folate	−0.32	−0.25	−0.27	0.03
Iron	−0.06	−0.19	−0.10	0.03
Saturated Fat	0.07	0.15	0.12	0.12
Sodium	−0.08	−0.26	−0.30	0.22
Vitamin A	−0.46 **	−0.18	−0.29	0.06
Vitamin B12	−0.04	−0.13	−0.01	0.29
Vitamin C	−0.43 **	−0.37 *	−0.40 *	0.17
Vitamin E	−0.30	−0.45 **	−0.29	0.01
Whole Grains	−0.06	−0.90	−0.08	0.12
Fruit	−0.34 *	−0.11	−0.13	0.14
Vegetable	−0.50 **	−0.27	−0.25	0.21

* *p* ≤ 0.05, ** *p* ≤ 0.01 based on Spearman rank order correlation coefficients.

**Table 6:** Comparisons of dietary intake variables by food security status.

Energy-Adjusted Dietary Intake	Food Security Status	*n*	U	*p*
Added Sugar	Secure	26	119.0	0.72
	Insecure	10		
Calcium	Secure Insecure	26	96.0	0.24
		10		
Fiber	Secure Insecure	26	126.0	0.90
		10		
Folate	Secure Insecure	26	124.0	0.85
		10		
Iron	Secure Insecure	26	95.0	0.23
		10		
Saturated Fat	Secure Insecure	26	93.0	0.20
		10		
Sodium	Secure Insecure	26	111.0	0.52
		10		
Vitamin A	Secure Insecure	26	78.0	0.07
		10		
Vitamin B12	Secure Insecure	26	104.0	0.37
		10		
Vitamin C	Secure Insecure	26	114.0	0.59
		10		
Vitamin E	Secure Insecure	26	128.0	0.96
		10		
Whole Grains	Secure Insecure	26	109.0	0.48
		10		
Fruit	Secure Insecure	26	109.0	0.48
		10		
Vegetable	Secure Insecure	26	84.0	0.11
		10		

## Data Availability

De-identified data can be made available upon request.
